# Phenological variation in biotic interactions shapes population dynamics and distribution in a range-shifting insect herbivore

**DOI:** 10.1098/rspb.2024.0529

**Published:** 2024-12-04

**Authors:** James E. Stewart, Ilya M. D. Maclean, Marc Botham, Emily B. Dennis, Jon Bridle, Robert J. Wilson

**Affiliations:** ^1^ College of Life and Environmental Sciences, University of Exeter, Exeter, UK; ^2^ Environment and Sustainability Institute, University of Exeter, Penryn Campus, Exeter, UK; ^3^ Centre for Ecology and Hydrology, Wallingford, Oxfordshire OX10 8BB, UK; ^4^ Butterfly Conservation, Manor Yard, East Lulworth, Wareham, Dorset, UK; ^5^ School of Biological Sciences, University of Bristol, Bristol, UK; ^6^ Department of Genetics, Evolution, and Environment, University College London, London, UK; ^7^ Departmento de Biogeografía y Cambio Global, Museo Nacional de Ciencias Naturales, Madrid E28006, Spain

**Keywords:** phenology, climate change, mechanism, trophic mismatch, population dynamics, range shift

## Abstract

Phenological responses to climate change vary across trophic levels. However, how trophic phenological synchrony determines species’ distributions through its effects on population dynamics has rarely been addressed. Here, we show that phenological variation underlies population and geographical range dynamics in a range-shifting herbivore, and demonstrate its interplay with changing trophic interactions. Using a novel modelling approach, we identify drivers of variation in phenology and population growth (productivity) for populations of the brown argus butterfly (*Aricia agestis*) feeding on ancestral and novel host plants in the UK. We demonstrate host plant-specific links between phenology and productivity, highlighting their role in the consumer’s range expansion. Critically, later butterfly phenology is associated with higher productivity in the annual second brood, especially on novel annual hosts where later activity improves synchrony with germinating plants. In turn, later phenology and higher second brood productivity are associated with more rapid range expansion, particularly in regions where only the novel hosts occur. Therefore, phenological asynchrony imposes limits on local population growth, influencing consumer resource selection, evolutionary responses and emergent range dynamics. How existing and future trophic phenological synchrony determine population dynamics will be critical for the ecological and evolutionary outcomes of climate change.

## 1. Introduction

Species’ geographical distributions have changed in extent, elevation and latitudinal range as populations track changing climates [1,2]. However, the magnitude and direction of terrestrial range shifts rarely match expectations based on rates of climate change [1,3,4]. Improved thermal suitability only permits expansion into areas with suitable resources (e.g. biotic interaction partners), and range expansion may be limited to areas with stable or increasing abundance [5–7]. As a result, the mechanisms underlying the effects of climate change on biotic interactions and local abundance are vital to understand range shifts and their consequences for ecological communities [8].

Alongside range shifts, phenological changes are among the best-documented responses to climate change [9–12], and are recognized for their effects on abundance and biotic interactions [13–19]. Fewer studies have explored the broader consequences of changing phenology for population dynamics or species’ distributions [15,20–22], although plant–insect interactions provide clear evidence for effects of phenological (a)synchrony on consumer demography [20,23–25]. Marked effects of plant–insect asynchrony are expected in interactions that are restricted to short time periods and require a high degree of synchrony [25–28]. Such interactions include insects feeding on plant tissues at specific phenological stages (e.g. orange-tip butterfly, *Anthocharis cardamines*, using flowers and seed pods of Brassicaceae hosts [29]), or those that must complete development rapidly before host senescence (e.g. Bay checkerspot butterfly, *Euphydryas editha bayensis*, feeding on *Plantago erecta* [30]).

We address the influence of phenological asynchrony on population dynamics and distribution in an exemplar range-expanding herbivore, the brown argus butterfly (*Aricia agestis*; Lepidoptera: Lycaenidae). Since the 1990s, the brown argus has undergone a climate-driven range expansion associated with rapid evolution of biotic interactions, including specialization on novel annual hosts (Geraniaceae species including *Erodium cicutarium*, *Geranium dissectum* and *Geranium molle*; Geraniales: Geraniaceae) in regions beyond its former range [31–36]. However, the condition and availability of these ephemeral hosts are more temporally variable than those of the perennial ancestral host plant *Helianthemum nummularium* (Malvales: Cistaceae; hereafter *Helianthemum*). This situation gives rise to a narrow and fluctuating phenological ‘window of opportunity’ to exploit the annual hosts, and may cause asynchrony between larvae and food availability [19], particularly in warmer, drier summers when the quality and recruitment of overwintering Geraniaceae hosts is lower [19]. Elucidating effects of climate-driven variation in host condition and phenological synchrony on brown argus population and range dynamics could offer insight into how eco-evolutionary dynamics shape climate stresses across species’ ranges, and thus how species and communities respond to environmental change [8,19,30,37,38].

Here, we test how the effects of climate on insect and host phenology influence insect population dynamics and range expansion. We employ a novel phenomenological modelling technique that estimates phenology, productivity (population growth) and abundance from count data [39]. We develop an extension to this approach to assess how the population dynamics of a specialist herbivore are linked to phenological synchrony in trophic interactions, related to host plant status and ecologically relevant fine-scale weather variables. We test for relationships between phenology and productivity, and assess how these vary between populations using different host plant species, to investigate how recent patterns of range expansion are driven by variable synchrony with novel annual host plants. We then consider whether population declines in butterfly populations specializing on the novel Geraniaceae hosts could arise via phenology shifts to earlier butterfly emergence. Observational and modelling work have previously highlighted that earlier emergence of the butterfly’s second annual brood can generate butterfly–host asynchrony due to egg-laying before annual germination of overwintering Geraniaceae hosts [19]. This system allows us to understand how phenological shifts can alter the outcomes of biotic interactions, with potentially transient effects on spatiotemporal persistence and structure of species’ ranges and ecological communities.

## 2. Methods

### (a) Study system

The brown argus’ UK geographic range was historically largely restricted to calcareous grassland where *Helianthemum* grows but has recently expanded alongside increased use of Geraniaceae host plants. Warming microclimates around annual Geraniaceae hosts appear to have enabled their increased use [32,40–42]. At recently colonized sites, brown argus females prefer Geraniaceae hosts (including *Erodium cicutarium*, *Geranium dissectum* and *G. molle*) for egg-laying, whereas at long-established sites, host preference tends to match the most common local host [31]. Females appear to select better condition leaves regardless of host species [31–34,40,42,43]. When laying on Geraniaceae, ovipositing females prefer younger, recently germinated hosts [42,44]. Given that condition and availability of the ephemeral Geraniaceae vary more over time and with (micro-)climatic conditions than those of *Helianthemum*, this interaction between host plant quality and female host preference creates spatial and temporal mosaics of availability and favourability for the consumer [19,42].

First-brood brown argus emerge in the UK from early May to late June. After egg-laying, their offspring develop and eclose as a second brood that flies between mid-July and mid-September. The offspring of this second brood feed on host leaf tissue during autumn, overwintering as larvae before resuming feeding, pupating and emerging as first-brood adults the following spring. Population growth rates are usually higher between first and second broods than over winter and, although larvae grow 10% larger and faster on Geraniaceae than on *Helianthemum*, poor overwinter productivity appears to be pronounced in populations feeding on Geraniaceae [39,41,43].

### (b) Brown argus distribution changes

We calculated the extent and rate of the brown argus’ range shift per decade for the period 1970−2018 using 10 km grid resolution (hectad) occurrence records from the National Biodiversity Network (NBN) [45] and the Global Biodiversity Information Facility [46] (electronic supplementary material, S1.1). First, we extracted the average northing values of the 10 northernmost (non-duplicate) hectad occurrence records for each decade and calculated the range expansion rate (km decade^−1^) as the increase in northing between the midpoint of each decade. We also quantified the extent of the brown argus’ UK range as the proportion of UK hectads occupied in each decade, and determined decadal range expansion as the change in this proportion over time.

Using NBN data [47], we assigned all UK hectads a host plant status: *Helianthemum* if the hectad contained a *Helianthemum* record, or Geraniaceae otherwise (electronic supplementary material, S1.1). Due to the widespread nature of the Geraniaceae, it is likely that some sites in ‘*Helianthemum* hectads’ also contain Geraniaceae species which may be used for egg laying; however, these are often limited growths in marginal areas whereas *Helianthemum* tends to be locally abundant with extensive ground cover where it is present.

For each year (1970−2018), we calculated interannual change in the proportion of occupied hectads where Geraniaceae were the only available hosts to indicate the year-on-year change in widespread use of Geraniaceae as a host. Finally, we calculated the interannual change in the number of occupied hectads to indicate the year-on-year change in range size.

### (c) Phenomenological modelling

#### (i) Phenomenological modelling: background

Dennis *et al*. [39] developed phenomenological models to estimate phenology (mean flight date: *μ*; length of flight period: *σ*) and productivity (population growth rate between adult generations: *ρ*) of butterflies from longitudinal count data, and derive abundance indices from estimates of productivity and initial abundance [39]. Here, we first outline the mathematical basis of the models when applied to univoltine (single-brooded) species, before describing its application to bivoltine species such as the brown argus. We then describe and apply an extension to these models, which allows for inter-brood stochasticity which may underpin some of the variation in counts, while simultaneously allowing up to two covariates to influence patterns of within-year (between-site) variation in counts.

Suppose butterfly counts are recorded at *S* sites, each visited on ≤*T* occasions in each of *Y* years (where *S* and *T* may vary between years). Each count represents the realization of a Poisson-distributed random variable with expectation λ*_i,j,k_* for site *i*, visit *j* and year *k.* For univoltine species, seasonal counts increase from zero and decrease to zero as adults emerge, fly and die. As is common in phenological studies [16,39], we assume the emergence period to approximate a normal probability density $N(\mu_{i,k},\sigma_{i,k}^{2})$ for site *i* and year *k*, so that for the *j*th site visit at time *t_i,j,k_,* we have


(2.1) $$\lambda_{i,j,k}=N_{i,k}\frac{1}{\sigma_{i,k}\sqrt{2\pi }}exp\left\{-\frac{{\left(t_{i,j,k}-\mu_{i,k}\right)}^{2}}{2\sigma_{i,k}^{2}}\right\},$$

which we write as $\lambda_{i,j,k}=N_{i,k}a_{i,j,k}$, where $N_{i,k}$ provides an estimate of relative abundance for a given site *i* and year *k*, and $a_{i,j,k}$ describes intra-annual seasonal variation over *j* visits to site *i*. Thus, the count for any visit has a Poisson distribution with a mean value proportional to the normal probability density function centred on $\mu_{i,k}$. Equation (2.1) may incorporate two normal distributions for application to bivoltine species:


(2.2) $$\begin{array}{ll} \lambda_{i,j,k} & =\;N_{i,k,1}\frac{1}{\sigma_{i,k,1}\sqrt{2\pi }}exp\left\{-\frac{{\left(t_{i,j,k}\;-\;\mu_{i,k,1}\right)}^{2}}{2\sigma_{i,k,1}^{2}}\right\}\\ & +N_{i,k,2}\frac{1}{\sigma_{i,k,2}\sqrt{2\pi }}exp\left\{-\frac{{\left(t_{i,j,k}\;-\;\mu_{i,k,2}\right)}^{2}}{2\sigma_{i,k,2}^{2}}\right\} \end{array}$$

Thus, for bivoltines, equation (2.2) may be written $\lambda_{i,j,k}\equiv \;N_{i,k,1}a_{i,j,k,1}+N_{i,k,2}a_{i,j,k,2}$. In each year, second brood abundance is related to that of the first via productivity terms (*ρ*), which represent conceptual products of the number of eggs laid by each adult, and their probability of contributing adults to the next generation. We therefore have $N_{i,k,2}={\rho_{i,k,1}N}_{i,k,1}$. Similarly, between-year dependence is given by $N_{i,k+1,1}={\rho_{i,k,2}N}_{i,k,2}$, where $\rho_{i,k,2}$ is the second brood productivity, which contributes to the relative abundance of the first brood in the following year ($N_{i,k+1,1}$). This recursion, developed over time, provides count estimates for each site, visit and year, predicated on an assumption that the relative abundance of a given brood depends on the abundance and productivity of the preceding brood. We therefore write


(2.3) $$\begin{array}{ll} \lambda_{i,j,1} & =N_{i,1,1}a_{i,j,1,1}+N_{i,1,2}a_{i,j,1,2}\\ & =N_{i,1,1}\left(a_{i,j,1,1}+\rho_{i,1,1}a_{i,j,1,2}\right) \end{array},$$

and


(2.4) $$\begin{array}{ll} \lambda_{i,j,k} & =\;N_{i,k,1}a_{i,j,k,1}+N_{i,k,2}a_{i,j,k,2}\\ & =\left(N_{i,1,1}\prod_{m=1}^{k-1}\prod_{b=1}^{2}\rho_{i,m,b}\right)a_{i,j,k,1}+\left(N_{i,1,1}\rho_{i,k,1}\prod_{m=1}^{k-1}\prod_{b=1}^{2}\rho_{i,m,b}\right)a_{i,j,k,2}\\ & =N_{i,1,1}\left(a_{i,j,k,1}+\rho_{i,k,1}a_{i,j,k,2}\right)\prod_{m=1}^{k-1}\prod_{b=1}^{2}\rho_{i,m,b}\;\text{for}\;k>1. \end{array}$$

#### (ii) Extending the phenomenological modelling approach

In the models presented in [39], *μ*, *σ* and *ρ* are estimated separately for each brood and each may either (i) remain constant across years, (ii) vary independently (non-linearly) with each year or (c) vary with up to two covariates (such as spatial and temporal variation in climatic conditions). Here, we extend the models to (d) allow *μ*, *σ* and *ρ* to vary simultaneously with both year and up to two covariates (combining and extending approaches (ii) and (iii) above; electronic supplementary material, S1.2). This extension allows elucidation of factors influencing changes in phenology and productivity. Schematic S1 (electronic supplementary material, S1.2) outlines the key elements of the phenomenological modelling approach. R code required to fit the models is provided in electronic supplementary material, S2.

### (d) Butterfly count data

We applied the models to brown argus count data obtained through the United Kingdom Butterfly Monitoring Scheme (UKBMS), based on weekly counts during favourable weather between early April and late September each year [48,49]. This gives a maximum of 26 visits (*j*) per site (*i*), per year (*k*), though our approach can account for variation in survey effort (number of visits) (electronic supplementary material, S1.2).

We categorized UKBMS sites at which brown argus had been recorded based on the main host plant type (Geraniaceae or *Helianthemum*) thought to be used on-site based on a multi-criteria assignment approach including data from sources such as site visits, flora lists and hectad-scale plant presence data (electronic supplementary material, S1.3). We then excluded sites for which fewer than five consecutive years of non-zero brown argus counts were available, leaving 80 Geraniaceae sites and 213 *Helianthemum* sites. We used data from these sites for the 25 year period 1992−2016, during which there were consistently more than five occupied sites per host plant per year. As outlined above, due to the widespread nature of the Geraniaceae, it is likely that some *Helianthemum* sites also contain Geraniaceae species which may be used for egg laying.

### (e) Covariates for phenomenological models

To determine drivers of phenology and productivity, we considered the following putative covariates: site northing and site- and year-specific mean and minimum daily overwinter temperature, temperature during the first brood (5 May–2 July), temperature during the period between the peaks of broods one and two (4 June–22 August; between-brood temperature, BBT), and mean daily temperature and rainfall in July, July–August, and in late summer (1 August–6 September; following [19]; electronic supplementary material, S1.4). Temperature and rainfall in late summer are important determinants of the condition and availability of Geraniaceae for the brown argus [19]. Overwinter temperatures refer to October–March (following [39]), and timings for brood-specific temperature data follow those estimated by Dennis et al. [39] (electronic supplementary material, S1.4). Temperature and rainfall data were derived from the UK Met Office 5 km gridded land surface daily observation dataset [50]. All covariates were standardized to have zero mean and unit variance.

### (f) Applying the phenomenological models

We first developed an all-sites model incorporating data from all 293 sites, to describe overall trends in productivity and phenology for each site, year and brood. In this simple model, productivity and phenology were permitted to vary year-to-year, and to vary within broods by site northing. We used Akaike’s information criterion (AIC) to assess performance against equivalent null models (electronic supplementary material, S1.5).

We then modelled productivity and phenology for populations using Geraniaceae or *Helianthemum* hosts. For each host type, we developed a global set of 210 models in which the key parameters describing productivity and phenology could vary according to scenarios a–d above. We then selected the most parsimonious (‘final’) model for each host type, to establish host-specific estimates of productivity and phenology (electronic supplementary material, S1.5 for model selection).

The models were fitted using a concentrated maximum likelihood approach (electronic supplementary material, S1.6) and verified based on performance when estimating parameters for simulated data (electronic supplementary material, S1.7 and table S1), following [39]. We derived abundance indices from estimates of productivity and initial abundance from the final model for each host and plotted these against year for each brood (electronic supplementary material, S1.8). We used generalized linear models (gamma errors, inverse link) and linear models, respectively, to test for differences in productivity and phenology (mean flight date) between hosts, and Spearman’s rank to assess directional trends in phenology over 1992−2016 (electronic supplementary material, S1.9).

### (g) Linking patterns in productivity and phenology

We extracted estimates of host-specific productivity and phenology (mean flight date and flight period length) from the final phenomenological models for each site and year, and tested for associations between these estimates. Model residuals demonstrated that it was not possible to fit acceptable models with site or year as random or fixed effects; we therefore averaged the productivity and phenology estimates (i) across years and (ii) across sites to give site-level and annual estimates of brood-specific productivity and phenology (for spatial and temporal analyses, respectively). All putative predictors (mean flight date, time between broods, mean flight period length and northing) were standardized (scaled and centred). Northing was included only where it was not identified as a predictor of *ρ* in the final phenomenological model for each host plant. We constructed candidate generalized linear models (gaussian or Gamma errors, identity, log or inverse link) by considering all plausible parameter combinations (including interactions and polynomial effects), estimated parameters using maximum likelihood and used AIC-based model selection to establish the most parsimonious model(s) for each host plant (electronic supplementary material, S1.10 for details of model selection, validation and diagnostics).

### (h) Linking distribution change to variation in productivity and phenology

Based on estimates from the final phenomenological models, we calculated phenology and productivity ‘anomalies’ representing the change in productivity or mean flight date from the 1992−2016 average for each brood and host plant (electronic supplementary material, S1.11). We used Spearman’s rank to test associations between these anomalies and (i) changes in Geraniaceae use (interannual change in the proportion of occupied hectads that were also Geraniaceae hectads) and (ii) changes in range size (interannual change in hectad occupancy).

## 3. Results

### (a) Brown argus distribution changes

**Figure 1.**
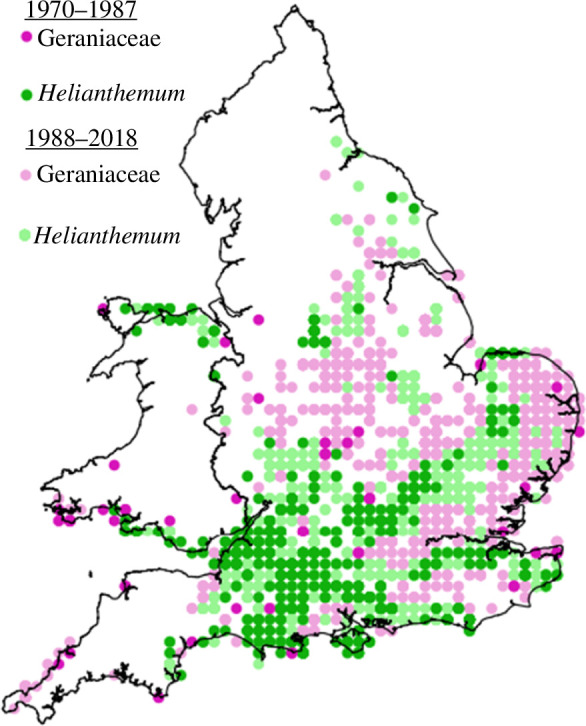
Brown argus occurrence in hectads containing *Helianthemum nummularium* (green) and Geraniaceae only (pink). Pre-expansion records (1970−1987) are in dark shades; hectads colonized during 1988−2018 are in light shades. Adapted and updated from [40].

The geographical range margin spread northwards by approximately 121 km between 1970−1979 and 2010−2018 (figure 1; average rate of 30.25 km decade^−1^, increasing in recent decades; electronic supplementary material, table S2). Hectad occupancy increased 4.2-fold over this period, with a disproportionate increase in hectads containing Geraniaceae compared with *Helianthemum* (electronic supplementary material, table S2 and figure S2). The increase in Geraniaceae hectad occupancy was not simply driven by increasing availability of these hectads during the range expansion (electronic supplementary material, S1.12).

Using outputs from the final phenomenological models for each host (PG_final_ and PH_final_, below), we found that the brown argus’ range increased following years of high first and second brood productivity at both Geraniaceae and *Helianthemum* sites, and in years following later second brood phenology at Geraniaceae sites (electronic supplementary material, S1.13). Its range dynamics were not associated with phenology at *Helianthemum* sites or first brood phenology at Geraniaceae sites (electronic supplementary material, S1.13). Brown argus occupancy of Geraniaceae hectads increased in following years with high second brood productivity (*ρ*_2_) and later second brood phenology (μ_2_) at Geraniaceae sites (Spearman’s *ρ*_*ρ*2_ = 0.459, *p* = 0.025; Spearman’s *ρ*_μ2_ = 0.498, *p* = 0.012) (electronic supplementary material, S1.13).

### (b) Productivity and phenology across broods and host plants

Productivity and phenology had significant year-to-year and within-year (between-site) variation associated with host plant type and environmental covariates. Across both broods, abundance was consistently higher at *Helianthemum* than Geraniaceae sites (electronic supplementary material, S1.14, figure S3).

**Figure 2.**
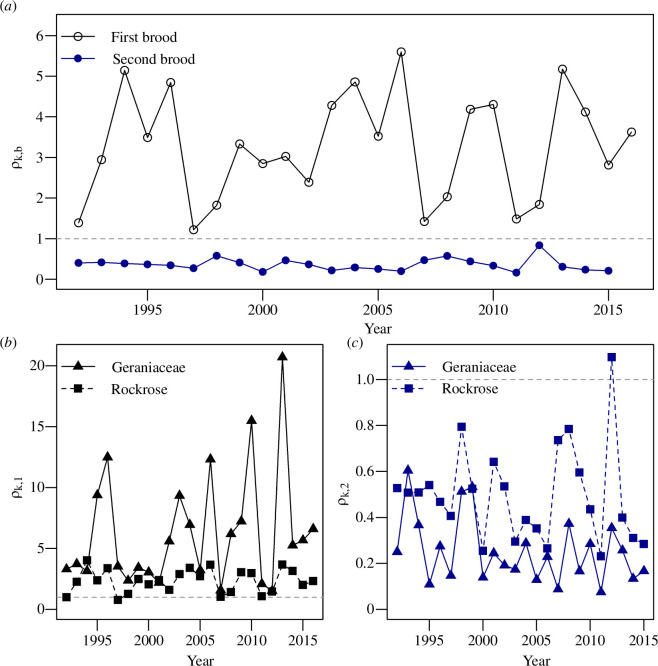
(*a*) Brown argus first and second brood productivities (*ρ*) from the all-sites model, plotted against year. (*b*) and (*c*) First and second brood productivities, respectively, at Geraniaceae and *Helianthemum* sites, from models PG_final_ (Geraniaceae sites) and PH_final_ (*Helianthemum* sites). *ρ* > 1 (dashed grey line) indicates population growth, *ρ* < 1 indicates decline.

Brown argus first brood productivity (summer population growth rate, *ρ*_1_) was consistently higher than second brood productivity (overwinter growth, *ρ*_2_) (all-sites model PA; table 1; figure 2*a*). Populations declined between the large second brood of year *k* and smaller first brood of year *k+1* (*ρ*_2_ < 1) (figure 2*a*). This annual bottleneck was stronger on Geraniaceae sites, which had significantly lower overwinter productivity than *Helianthemum* sites (figure 2*c*; electronic supplementary material, S1.15) but significantly higher summer productivity (figure 2*b*; electronic supplementary material, S1.15).

**Table 1.** Summary of AIC analysis for all-sites (PA), Geraniaceae sites (PG) and *Helianthemum* sites (PH) phenomenological models, with null models (_null_) for comparison, showing slopes of productivity (*ρ*) and phenology (mean flight date, *μ*) on site northing, between-brood temperature (BBT), July–August rain (JAR), late summer temperature (LST), overwinter (October–March) temperature leading into the focal year *k* (OWT−) and overwinter temperature leading into year *k+1* (OWT+). Also showing the mean (±s.d.) of the intercept for these slopes across years (based on year-to-year variation in productivity and phenology), all on the log scale, and the mean productivity and phenology (across sites and years) for each brood (*ρ*_*k*,1_, *ρ*_*k*,2_, *μ*_*k*,1_ and *μ*_*k*,2_). Standard errors of slope estimates are presented in parentheses, based on the model Hessian matrix. LL, log-likelihood; *k*, number of parameters; ΔAIC, change in AIC relative to lowest-AIC model (denoted ‘_best_’). The final selected model in each case is denoted ‘_final_’.

model	*ρ*_1_ estimates					*ρ*_2_ estimates						*μ*_1_ estimates				*μ*_2_ estimates				*LL*	*k*	ΔAIC
	mean ρ_k,1_	int. (mean ± SD)	slope: north	slope: BBT	slope: OWT−	mean *ρ*_*k*,2_	int. (mean ± s.d.)	slope: north	slope: JAR	slope: LST	slope: OWT+	mean μ_k,1_	int. (mean ± s.d.)	slope: north	slope: OWT−	mean *μ*_*k*,2_	int. (mean ± s.d.)	slope: north	slope: BBT			
**PA_final_**	2.749	0.908 (0.463)	0.134 (0.008)	–	–	0.435	−0.920 (0.406)	−0.127 (0.008)	–	–	–	9.239	–	–	–	20.520	–	–	–	−121748.3	151	0.00
**PA_null_**	2.476	–	–	–	–	0.400	–	–	–	–	–	8.957	–	–	–	20.437	–	–	–	−142250.7	6	24372.74
**PG_best_**	6.250	1.588 (0.707)	0.019 (0.002)	−0.034 (0.006)	–	0.249	−1.538 (0.552)	–	0.049 (0.005)	0.083 (0.006)	–	9.272	2.221 (0.108)	0.017 (0.004)	0.005 (0.001)	19.807	2.352 (0.077)	−0.019 (0.004)	−0.019 (0.004)	−22876.25	161	0.00
**PG_final_**	6.262	1.590 (0.707)	0.019 (0.002)	−0.034 (0.006)	–	0.249	−1.540 (0.550)	–	0.049 (0.005)	0.083 (0.006)	–	9.273	2.222 (0.108)	0.013 (0.004)	–	19.808	2.352 (0.076)	−0.018 (0.004)	−0.017 (0.003)	−22877.36	160	0.23
**PG_null_**	4.979	–	–	–	–	0.206	–	–	–	–	–	8.888	–	–	–	19.600	–	–	–	−26752.47	6	7442.44
**PH_final_**	2.471	0.801 (0.521)	–	–	−0.241 (0.012)	0.476	−0.828 (0.517)	–	–	–	0.225 (0.010)	9.346	2.228 (0.117)	0.044 (0.001)	–	20.631	2.419 (0.075)	−0.055 (0.002)	–	−94444.9	155	0.00
**PH_null_**	2.260	–	–	–	–	0.435	–	–	–	–	–	8.965	–	–	–	20.593	–	–	–	−113349.2	6	37510.6

The main environmental correlates of productivity differed between populations using the two host types. At *Helianthemum* sites, summer population growth was higher after colder winters (table 1; electronic supplementary material, figure S4a), and overwinter population growth was positively associated with overwintering temperature (table 1; electronic supplementary material, figure S4b). By contrast, overwinter population growth at Geraniaceae sites was positively associated with July–August rainfall and late summer temperature (table 1; electronic supplementary material, figure S5c,d), whereas summer population growth was higher further north but lower when the between-brood (June–July) temperature was higher (table 1; electronic supplementary material, figure S5a,b).

Neither first nor second brood phenology advanced over the period 1992−2016 (electronic supplementary material, S1.16, figure S6), but there was significant year-to-year variation in first and second brood mean flight dates (*μ*_b_) at both Geraniaceae and *Helianthemum* sites (table 1). First brood phenology did not differ between host plants (electronic supplementary material, figure S6b and table S4), but second brood phenology was approximately 6 days earlier at Geraniaceae than *Helianthemum* sites (electronic supplementary material, figure S6c; electronic supplementary material, table S4). Within years, *μ*_1_ was later further north on both hosts (table 1; electronic supplementary material, figures S7a and 8a). In contrast, *μ*_2_ was *earlier* further north on both hosts (table 1; electronic supplementary material, figures S7b and 8b). At Geraniaceae sites, *μ*_2_ was also earlier following warmer summers (when the BBT was higher; table 1; electronic supplementary material, figure S8c).

### (c) Links between phenology and productivity

**Figure 3.**
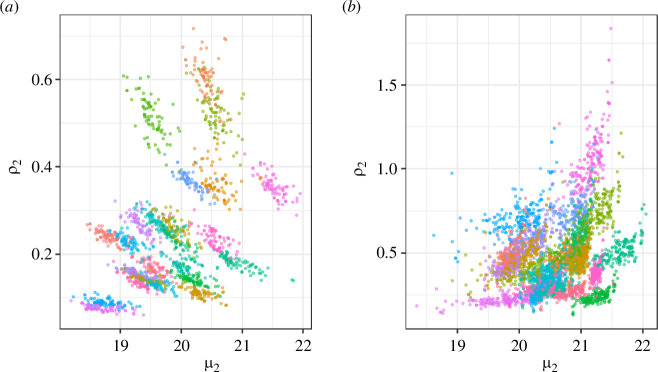
Second brood productivity (*ρ*_2_) against second brood mean flight date (*μ*_2_; week number from 1 April) for brown argus at (*a*) Geraniaceae, and (*b*) *Helianthemum* sites. Each point represents a yearly estimate for each of (*a*) 80 and (*b*) 213 sites. Years are shown in different colours to allow visualization of both within-year and among-year changes in productivity (*ρ*).

**Figure 4.**
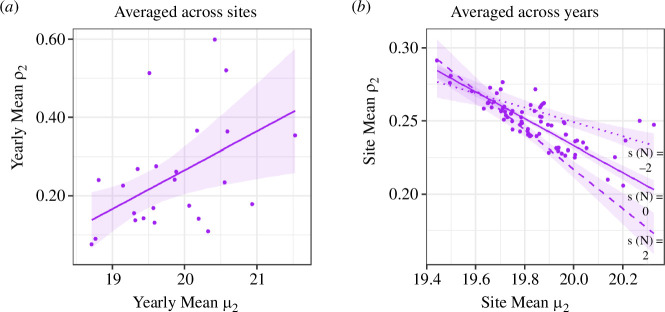
Relationships (with 95% confidence intervals) between second brood phenology (mean flight date, *µ*_2_, in weeks since 1 April), northing (standardized, *s*(N)) and second brood productivity (mean overwinter population growth, *ρ*_2_) at Geraniaceae sites. Estimates derived from models (*a*) G-*ρ*_2-tm-final_ and (*b*) G-*ρ*_2-sp-final_ (table 2). In (*a*) point estimates represent the mean across years at individual sites; while point estimates in (*b*) represent the average across sites in each of 24 years. NB. Axis limits differ between plots.

The links between brown argus phenology and productivity depended on the brood (electronic supplementary material, S1.17), host plant (figure 3), and whether considered across sites or across years (figures 3 and 4). Averaged across Geraniaceae sites, the second generation had higher overwinter productivity during years in which they emerged later (table 2a; figure 4*a*). By contrast, when estimates were averaged across years, second brood productivity was lower at Geraniaceae sites with later second brood emergence, with a weak effect of site northing (table 2b; figure 4*b*). These contrasting relationships between sites and years generate the distinct patterns indicated in figure 3 (summarized in figure 4*a,b*). There was also limited evidence that years with longer second-generation flight periods had lower productivity at Geraniaceae sites (table 2a). First brood phenology was not related to productivity at Geraniaceae sites; in contrast, averaged across *Helianthemum* sites, first brood brown argus were more productive in years when mean flight date was earlier (electronic supplementary material, S1.17).

**Table 2.** Summary of AIC analyses for generalized linear models of drivers of brown argus second brood productivities (*ρ*_2_) at Geraniaceae (G) and *Helianthemum* (H) sites. The selected model is denoted ‘_final_’ and the null model (_null_) is presented for comparison. Models are presented for data averaged across sites (temporal patterns, _tm_) and across years (spatial patterns, _sp_). Parameter notation: ‘μ_2_’ is the mean flight date of the second brood, ‘σ_2_’ is the length of the second brood flight period. LL, model log-likelihood.

model	parameter estimates (and standard errors)							*LL*	AIC
	intercept	*μ*_2_	(*μ*_2_)^2^	(*μ*_2_)^3^	site northing	*μ*_2_: northing	σ_2_		
(*a*) Geraniaceae sites: **temporal** patterns (site-averaged). Gamma errors (inverse link).									
**G-ρ_2-tm-final_**	4.226 (0.468)	−0.871 (0.393)	–	–	–	–	–	19.63	−33.27
**G-ρ_2-tm-sigma_**	4.229 (0.474)	–	–	–	–	–	1.008 (0.518)	19.44	−32.88
**G-ρ_2-tm-null_**	4.024 (0.466)	–	–	–	–	–	–	17.14	−30.28
(*b*) Geraniaceae sites: **spatial** patterns (year-averaged). Gaussian errors (identity link).									
**G-ρ_2-sp-final_**	0.248 (0.001)	−0.016 (0.001)	–	–	−0.005 (0.001)	−0.004 (0.001)	–	276.63	−543.26
**G-ρ_2-sp-null_**	0.249 (0.002)	–	–	–	–	–	–	217.29	−430.58
(*c*) Helianthemum sites: **spatial** patterns (year-averaged). Gamma errors (inverse link).									
**H-ρ_2-sp-final_**	2.116 (0.015)	−2.586 (0.228)	−0.368 (0.229)	−0.820 (0.220)	–		–	346.51	−683.02
**H-ρ_2-sp-null_**	2.099 (0.020)	–	–	–	–		–	281.65	−559.31
(*d*) Helianthemum sites: **temporal** patterns (site-averaged). No models outperformed the null. Gamma (identity).									
**H-ρ_2-tm-null_**	0.477 (0.040)	–	–	–	–	–	–	7.67	−11.344

**Figure 5.**
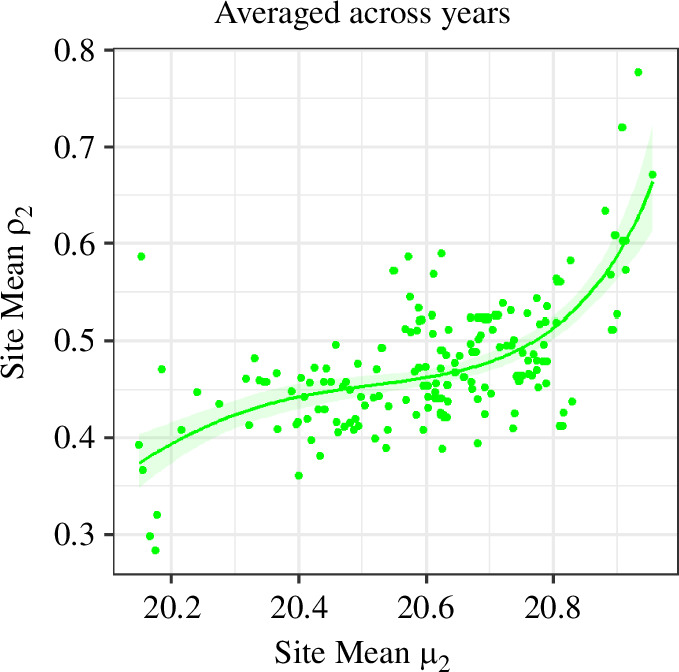
Relationship (with 95% confidence intervals) between second brood phenology (mean flight date, *µ*_2_, in weeks since 1st April), and second brood productivity (mean overwinter population growth, *ρ*_2_) at *Helianthemum* sites. Estimates derived from model H-*ρ*_2-sp-final_ (table 2). Point estimates represent the mean across years at individual sites.

Considering *Helianthemum* sites averaged across years, second brood productivity (overwinter population growth) was higher at later-emerging *Helianthemum* sites (table 2c, figure 5). Interannual variation in second brood phenology was not associated with interannual differences in second brood productivity at *Helianthemum* sites (table 2d).

### (d) Drivers of brown argus distribution change

The proportion of brown argus records in Geraniaceae hectads increased following years with high second brood productivity (*ρ*_2_) and later second brood flight periods (*μ*_2_) on Geraniaceae sites (Spearman’s *ρ*_ρ2_ = 0.459, *p* = 0.025; Spearman’s *ρ*_μ2_ = 0.498, *p* = 0.012), but not following years of high *ρ*_2_ or later *μ*_2_ on *Helianthemum* sites (Spearman’s *ρ*_ρ2_ = 0.308, *p* = 0.143; Spearman’s *ρ*_μ2_ = 0.260, *p* = 0.209). The change in the proportion of brown argus records in Geraniaceae hectads was not associated with high *ρ*_1_ or later *μ*_1_ in the current or previous year at Geraniaceae sites or *Helianthemum* sites (absolute value of Spearman’s *ρ* < 0.366, *p* > 0.05 in all cases).

The overall number of hectads occupied by brown argus increased following years with high second brood productivity at both Geraniaceae (G) and *Helianthemum* (H) sites (Spearman’s *ρ*_G_ = 0.458, *p* = 0.025; Spearman’s *ρ*_H_ = 0.565, *p* = 0.005), and also increased in years following later second brood emergence at Geraniaceae sites (Spearman’s *ρ*_G_ = 0.498, *p* = 0.012), but not following later second brood emergence at *Helianthemum* sites (Spearman’s *ρ*_H_ = 0.392, *p* = 0.053). The overall number of hectads occupied by brown argus also increased in years with high first brood productivity at both Geraniaceae sites and *Helianthemum* sites (Spearman’s *ρ*_G_ = 0.634, *p* = 0.001; Spearman’s *ρ*_H_ = 0.578, *p* = 0.004), but was not associated with the timing of first brood emergence at either *Helianthemum* or Geraniaceae sites (Spearman’s *ρ*_G_ = 0.248, *p* = 0.230; Spearman’s *ρ*_H_ = 0.280, *p* = 0.175).

## 4. Discussion

Until recently (e.g. [12,14,16]), few studies have explicitly considered the consequences of variation in phenology for population dynamics, and fewer have explored subsequent impacts on species’ ranges [15]. We demonstrate brood- and host plant-specific links between climate, phenology and population dynamics in a range-expanding species, outlining how climate change may influence the abundance and geographical distribution of a species through its effects on the relative phenology of a biotic interaction. Later phenology at the end of the temperate growing season correlates with larger population size the following spring, especially on annual hosts where later butterfly activity may enable greater larval synchrony with autumn-germinating plants. The potential bottlenecks that can be imposed on population growth by phenological changes that generate consumer-resource asynchrony, may therefore be important determinants of the outcomes of climate change for species range shifts.

### (a) Linking phenology and population dynamics

Productivity of brown argus populations using an ancestral, perennial host (*Helianthemum*) depended on overwinter temperatures, which is consistent with a multi-species study in which brown argus abundance declined significantly between years in association with extreme winter cold [51]. By contrast, first brood productivity in populations using annual hosts (Geraniaceae) was greater when the temperature during the period between the first and second brood was lower, and at higher latitudes. These drivers may be linked (temperatures tend to be lower further north in the UK), and may relate to the condition of the novel Geraniaceae hosts, which have been shown to senesce rapidly in warm summers [19].

Second brood productivity at Geraniaceae sites was higher following wetter conditions over July and August, and was promoted by warmer late summer temperatures. Wet summer conditions could enhance germination and growth of the ephemeral annual Geraniaceae hosts (as suggested following modelling based on measurements of wild Geraniaceae in [19]), providing high-quality food for offspring of the second brood. Warmer late summer temperatures may enhance productivity by encouraging flight and mating behaviours, or by hastening larval development. Thus, the climatic factors influencing consumer population growth vary both over time (within years) and depending on the host plant species used.

Many studies have shown positive demographic responses to advancing phenology associated with climate change [13,15,21]. In contrast, in our study, interannual phenological advances and broader flight periods were associated with lower productivity in second brood brown argus at Geraniaceae sites (figures 3 and 4*a*). At Geraniaceae sites, young, high-quality hosts emerge following late summer/early autumn rains and are strongly preferred for egg-laying by second brood females when compared to older, poorer-quality Geraniaceae [19,42]. By increasing the synchrony between suitable host plants and key butterfly life stages (e.g. adult egg-laying and early larval stages), late butterfly emergence may encourage oviposition on suitable hosts and increase larval survival.

Figures 3 and 4*b* indicate another correlation between second brood phenology and productivity: at sites where the second brood tends to emerge later, overwinter population growth tends to be lower. This is in part explained by effects of site northing (figure 4*b*). However, there is relatively little spatial variation in year-averaged emergence date (less than one week; figure 4*b*), and the effect size is relatively small, for example, compared with that depicted in figure 4*a*.

There was no observed relationship between site-averaged phenology and productivity in second brood brown argus at *Helianthemum* sites; while on average across years, later-emerging *Helianthemum* sites tended to be slightly more productive in the second generation. Plant quality and availability are locally less variable in *Helianthemum* than in the Geraniaceae hosts [19], though moisture levels can affect nitrogen bioavailability [33,52], with consequences for both larval growth and myrmecophily [53,54].

### (b) Consequences for range expansion

Our results show how variation in herbivore phenology can alter the synchrony of trophic interactions, with consequences for population dynamics and distribution. Specifically, our data suggest that late second brood emergence at Geraniaceae sites increases the synchrony of egg-laying and larval emergence with availability of high-quality annual host plants [19]. This likely promotes larval growth and development, encouraging population growth and range expansion. As a result, brown argus range expansion through landscapes containing the novel Geraniaceae host plants is faster during years with later second brood emergence: this coincidence of late second brood emergence with availability of germinating plants appears to be an important motor of the recent range expansion and selection for egg-laying on the novel hosts [19,36,42].

However, brown argus second brood phenology advances by approximately 2 days per degree of warming at Geraniaceae sites (table 1). If future warming and phenological plasticity result in widespread advancement of second brood phenology at Geraniaceae sites, this may generate further asynchrony with Geraniaceae hosts that emerge later following warm and dry summers [19]. Late-summer quality and seedling recruitment of overwintering Geraniaceae hosts are poorer with higher summer temperatures and low late-summer rainfall [19]. Based on our data, this could stall or reverse the recent range expansion as populations at Geraniaceae sites decline. The likelihood of this situation will also depend on, for example, the influence of microclimatic heterogeneity on local patterns of host quality, phenology and biotic interactions; intra-specific variation in resource and consumer sensitivity to phenological cues; and the potential for consumer evolutionary responses [20,42,55]. For example, selective pressures from phenological mismatches triggered an evolutionary response towards delayed hatching time in the winter moth *Operophtera brumata*, decreasing asynchrony with resource availability [20].

Between 1970−1979 and 2010−2018, the brown argus expanded its range northwards by an average of 30.25 km decade^−1^, and by 70 km decade^−1^ between the periods 2000−2009 and 2010−2018. The recent expansion rate is much faster than the average of range shifting species (e.g. 16.9−23.2 km decade^−1^ [2,56], and has been associated with increased use of Geraniaceae (this paper, [41]) and an increase in the spatial scale of adaptation [31]. Specifically, selection has apparently favoured more dispersive phenotypes which have increased flight capacity and more readily accept the widespread Geraniaceae hosts [31,32,40]. Consequently, the dispersive, Geraniaceae-favouring phenotype may represent an alternative life history strategy that drives expansion at range margins and in-filling of the core range. Subsequent migrants that colonize *Helianthemum* sites may need to regain the ability to use *Helianthemum* (as shown in [34]) in order to benefit from stability of (and phenological synchrony with) the host resource [19].

### (c) Broader implications

These results provide evidence for important relationships between phenology, population dynamics and distribution, exemplified by a specialist herbivorous insect. The ability of consumers to synchronize feeding with the appropriate stage of plant development is a key aspect of widespread plant–herbivore interactions, and can have a major impact on herbivore performance and population dynamics [29,57–59]. In our case, we made the interesting observation that population growth on the ancestral host appears to be limited by cold winter temperatures, which might be expected at the high-latitude range margin of a species. In contrast, population growth on the novel host was promoted by relatively cool midsummer temperatures between the two annual broods, linked to delayed emergence of the butterfly’s second brood, and warm wet conditions in late summer which promote Geraniaceae host germination and quality [19]. Consequently, to fully understand consumer population dynamics and conservation status, it will be important to identify the factors that underlie both their phenology and that of their resources. This is especially true for species in which phenological shifts are expected to be detrimental: those with short interaction windows, including species that utilize a limited number of ephemeral resources [25–28,59].

However, understanding phenology-mediated climate impacts on consumer population dynamics and distributions is likely to be challenging for most species, since it necessitates repeated monitoring over multiple broods at single sites, preferably replicated across space. Although statistical approaches are emerging that can deal with many aspects of these complex systems [14,39,60,61], such data are rare, and biased towards early spring phenological responses in a few taxa across a few regions (e.g. insects and birds in northwest Europe and North America [10,13,60]). Such limitations prevent a full understanding of the mechanisms by which climate influences population dynamics and species distributions, and restrict our ability to predict future responses to environmental change and to mitigate impacts. Given these uncertainties and the high degree of inter-specific variation in climate change responses, conservation could seek to maximize opportunities for synchrony in interspecific interactions. For example, enhancing habitat and microclimatic heterogeneity can buffer populations against trophic mismatch and population decline by locally maximizing the temporal window of opportunity for trophic interaction [15,62–64].

## 5. Conclusions

Few studies have addressed the links between phenology, population dynamics and geographical distributions, or their fate under a variable or changing climate [15,21]. Here, we show that phenological variation underlies patterns of distribution change via effects on population dynamics, and demonstrate how this may be mediated by differences in the strength of biotic interactions among host species. Such relationships are underexplored but may be widespread, as many species vary in their resource use and specificity over space and time [15,29,65]. This work highlights the importance of accounting for trophic synchrony and intraspecific variation in resource use when investigating phenology and population dynamics, species distributions, and the wider consequences for ecological communities.

## Data Availability

The electronic supplementary material and datasets supporting this article are available online [66].
